# Turbulence-Resilient Object Classification in Remote Sensing Using a Single-Pixel Image-Free Approach

**DOI:** 10.3390/s25134137

**Published:** 2025-07-02

**Authors:** Yin Cheng, Yusen Liao, Jun Ke

**Affiliations:** 1 School of Optics and Photonics, Beijing Institute of Technology, Beijing 100081, China; 3120225310@bit.edu.cn (Y.C.);; 2 Key Laboratory of Photo-Electronic Imaging Technology and System, Ministry of Education of China, Beijing 100081, China; 3 National Key Laboratory on Near-Surface Detection, Beijing 100072, China

**Keywords:** object classification, image processing, single pixel imaging, atmospheric turbulence

## Abstract

In remote sensing, object classification often suffers from severe degradation caused by atmospheric turbulence and low-signal conditions. Traditional image reconstruction approaches are computationally expensive and fragile under such conditions. In this work, we propose a novel image-free classification framework using single-pixel imaging (SPI), which directly classifies targets from 1D measurements without reconstructing the image. A learnable sampling matrix is introduced for structured light modulation, and a hybrid CNN-Transformer network (Hybrid-CTNet) is employed for robust feature extraction. To enhance resilience against turbulence and enable efficient deployment, we design a (N+1)×L hybrid strategy that integrates convolutional and Transformer blocks in every stage. Extensive simulations and optical experiments validate the effectiveness of our approach under various turbulence intensities and sampling rates as low as 1%. Compared with existing image-based and image-free methods, our model achieves superior performance in classification accuracy, computational efficiency, and robustness, which is important for potential low-resource real-time remote sensing applications.

## 1. Introduction

Although deep learning has revolutionized remote sensing image analysis through data-driven feature representation, critical challenges still persist, including atmospheric turbulence-induced distortions, sensor noise contamination, and dynamic occlusion scenarios, which substantially degrade the classification robustness of computer vision models in real-world environments [1]. Among these challenges, the complex imaging process of turbulence aberrations involves multiple spatio-temporally varying geometric aberrations and blurring states, stemming from disorder in the light propagation medium. On the other hand, one of the most promising applications of single-pixel imaging (SPI) is remote sensing. Single-pixel imaging (SPI) is a computational imaging technique that reconstructs two-dimensional (2D) images from one-dimensional (1D) measurements based on the principle of correlation [2]. Over the years, approaches based on compressed sensing (CS) [3,4,5] and compressed learning (CL) [6] have enabled high-quality image reconstruction with reduced measurements. In 2010, Han’s team investigated the impact of atmospheric turbulence on GI (ghost imaging) performance and demonstrated its turbulence-mitigating capabilities [7]. Later, Erkmen studied the application of GI in remote sensing systems and quantitatively analyzed the influence of non-uniform turbulent distribution on GI [8]. Although conventional two-path GI methods are capable of obtaining images that are turbulence-free [9,10], addressing the degradation of the quality of SPI in turbulent environments remains a challenge [11].

With the development of computer vision, object classification using SPI has been applied in various fields. However, the traditional imaging before perception method relies on extracting features from reconstructed images [12,13], and its computing cost, operating efficiency, and data transmission all imply higher requirements for hardware resources and reconstruction algorithms. Recently, the concept of image-free computational perception was proposed. Without complex measurement processes, image-free semantic reasoning can realize action recognition and face recognition with a high compression rate [14,15]. The image-free learning technology based on the convolutional neural network has achieved higher accuracy in image recognition datasets such as MNIST, fashion MNIST, and CIFAR-10 [16,17,18].

Despite many existing methods for imaging with atmospheric turbulence or methods for object classification without atmospheric turbulence, there is a lack of approaches that specifically address object classification for long-range remote sensing in turbulence-degraded environments without imaging. To address this gap, we propose an end-to-end classification method based on single-pixel imaging (SPI) and deep learning, which combines a learnable sampling matrix with a hybrid CNN-Transformer network (Hybrid-CTNet) for the first time. The architecture of the system consists of an encoding module (structured light modulation and single-pixel detection) and a classification module (Hybrid-CTNet). End-to-end optimization is employed to achieve efficient feature extraction. Unlike prior work that either uses fixed sampling matrices (e.g., Hadamard, random, PCA-based) or separates sampling from inference, our method jointly optimizes the sampling matrix and classification backbone in a fully end-to-end manner. This allows the sampling patterns to adapt to both scene semantics and environmental distortions such as atmospheric turbulence, resulting in more informative and robust 1D measurements. Moreover, in contrast to reconstruction-based pipelines, our approach performs direct classification from compressed measurements, entirely bypassing image reconstruction, which reduces computational cost and improves efficiency.

Compared to existing methods, this framework maintains high robustness at low sampling rates (3%) and strong turbulence (100 layers), achieving state-of-the-art classification performance on benchmark datasets and in real-world optical experiments. These contributions make the proposed system well-suited for lightweight deployment in practical remote sensing scenarios.

## 2. Related Work

SPI is a promising method for remote sensing applications, allowing high-quality imaging with minimal equipment for Earth observation, atmospheric monitoring, and space exploration. However, atmospheric turbulence distorts light propagation, challenging SPI systems.

Recent advances in deep learning, particularly transformer networks and generative models, have significantly improved SPI by suppressing distortions caused by turbulence, allowing high-quality imaging in turbulent environments [19,20], because neural networks have proven to be effective in handling complex non-linear problems, thus being able to capture the underlying structure of turbulent evolution through their parameters and activation functions. Lyu et al. first applied neural networks to ghost imaging (GI) reconstruction in 2017 [21], followed by He et al. in 2018, who extended this approach to grayscale image reconstruction [22]. In 2021, Zhang and Duan utilized multiscale generative adversarial networks for CGI reconstruction under turbulence [23], while Zhang, Bian et al. processed Fourier coefficients in Fourier single-pixel imaging to filter noise and employed GANs to mitigate turbulence effects [24]. More recently, Zhang and Zhai proposed an end-to-end imaging method for the suppression of turbulence in 2023 [25].

**Figure 1:**
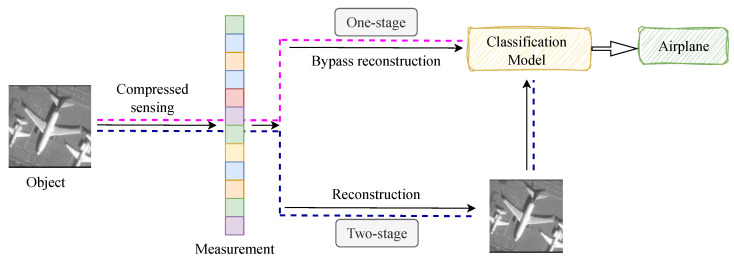
Two methods of compressed sensing object classification.

SPI-based object classification includes image-based and image-free approaches. The former reconstructs images before classification, while the latter directly infers object classes from raw measurements. As shown in Figure 1, the lower branch follows the traditional two-stage process, while the upper branch adopts the emerging image-free paradigm. Wang et al. [26] proposed a single-stage end-to-end network trained on simulated data, achieving image reconstruction without modulation patterns and attaining 92% classification accuracy in MNIST at a 6.25% sampling rate. Li et al. [27] improved classification efficiency by incorporating measurement values and Hadamard modulation patterns into classification networks, reducing processing time while improving accuracy. Yang et al. [28] developed a multitask learning framework capable of performing image reconstruction, object classification, and localization simultaneously. Pan et al. [29] utilized mask-based lensless optics and Transformers for incoherent non-reconstruction object recognition. In contrast, image-free classification streamlines the process by directly entering 1D measurements into classification models [30]. Bian et al. [31] employed CRNNs (Convolutional Recurrent Neural Networks) with bidirectional LSTMs (Long Short-Term Memory) for license plate detection, achieving 87.60% accuracy at a sampling rate 5% and 100+ FPS. Peng et al. [32] developed a non-imaging single-pixel object detection network using Transformers, achieving 82.41% mAP at a 5% sampling rate and 63 FPS. He et al. [33] explored shallow learning in SPIF (Single-Pixel Image-Free), applying PCA (Principal Component Analysis) to extract features and effectively reducing training time to optimize the deep-learning-based measurement matrix. For classification tasks in challenging environments such as atmospheric turbulence, Liao et al. [34] proposed a dual-task learning approach for long-range classification using single-pixel imaging.

As discussed above, recent studies have shown significant progress in nonimaging object classification using SPI. However, challenges remain with complex natural scenes and turbulent environments. On the other hand, recent efforts have focused on integrating Convolutional Neural Networks (CNNs) and Transformers for efficient deployment. Traditionally, methods employ convolutional blocks in early stages and stack Transformer blocks towards the end. However, this hybrid approach often reaches performance saturation in downstream tasks, such as segmentation and detection, as it may not capture comprehensive global information from all layers. This paper presents a novel transformer-based method for single-pixel object classification. This study presents a Hybrid-CTNet for single-pixel object classification, integrating deep learning with single-pixel imaging for direct inference from minimal measurements. Structured light modulates object information, which is sampled using a learnable single-pixel measurement matrix that is jointly optimized with the neural network. Additionally, an innovative (N+1)×L hybrid strategy is proposed, where Transformer blocks are strategically placed after N convolutional blocks in each stage, enhancing global feature extraction from shallow layers to improve overall performance and deployment efficiency. This approach is highly resilient to turbulence and adaptable at varying sampling rates, achieving robust performance even at 1%. Validation in public datasets and optical experiments underscores its effectiveness, particularly advantageous for lightweight hardware and software implementations.

## 3. Turbulence-Resilient Classification Using SPI with Hybrid-CTNet

**Figure 2:**
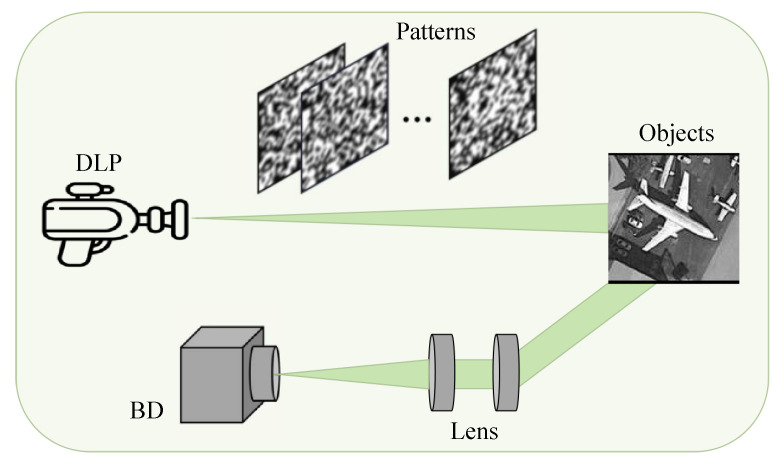
Diagram of single pixel imaging principle.

To improve data acquisition and classification efficiency in remote sensing, we propose an end-to-end classification framework integrating single-pixel imaging (SPI). Our approach is reconstruction-free with the measurement matrix co-optimized by the inference network. The system consists of two main modules: the encoding module and the classification module. As shown in Figure 2, the encoding module compresses the optical field of the object into one-dimensional measurements using a learnable sampling matrix (Equation (3)) and simulates atmospheric turbulence via a phase screen model (Equations (4) and (5)). This effectively captures essential scene features in a compressed form, reducing data load while preserving necessary classification information. The classification module directly processes these one-dimensional measurements using a deep neural network trained to recognize patterns without requiring full image reconstruction. It employs the (N+1)×L hybrid strategy, stacking N convolutional blocks followed by one Transformer block per stage. By integrating frequency-domain attention and efficient multi-head self-attention, the system enables local-global feature co-modeling and multi-scale fusion, ensuring fast and accurate classification, which is particularly beneficial for resource-limited remote sensing applications.

### 3.1. Single-Pixel Imaging for Atmospheric Turbulence

The process of single-pixel imaging (SPI) begins with the projection of structured light patterns, such as speckle fields, onto the object scene. This structured light serves to encode the spatial information of the scene onto a one-dimensional measurement, which is captured by a single-pixel detector. Unlike traditional imaging systems, which use arrays of detectors to directly capture spatial data, SPI relies on capturing the total light intensity reflected or transmitted from the scene. This approach drastically reduces the dimensionality of the data while retaining essential features of the object, making the process much more data efficient, especially in low-resolution and resource-constrained environments.

This data reduction, while maintaining vital scene information, is achieved through the correlation of the structured light with the object. The one-dimensional measurement value coupled with the light intensity received by the single-pixel detector can be expressed mathematically as

(1) $$\begin{array}{c} B_{i}=\;\int \int \;I_{i}\left(x,y\right)O_{i}\left(x,y\right)dxdy \end{array}$$

where $I_{i}\left(x,y\right)$ represents the intensity of the speckle field at each point and $O_{i}\left(x,y\right)$ denotes the object or target to be measured at each corresponding location. The index *i* represents the number of sampling times or measurements taken by the single-pixel detector. These measurements essentially capture the interaction between the object scene and the structured light pattern, translating the complex scene into a compressed one-dimensional form.

During target reconstruction, the measurements obtained by the detector are used in conjunction with known light templates to assess the correlation with the intensity data. This process allows for the reconstruction of the target object, and it can be mathematically described by

(2) $$\begin{array}{c} G\left(x,y\right)=\langle B_{i}I_{i}\left(x,y\right)\rangle -\langle B_{i}\rangle \langle I_{i}\left(x,y\right)\rangle \end{array}$$

where 〈·〉 represents the measurement averaging operation. This step effectively reconstructs the target’s spatial information by isolating the relevant features from the combined measurement data. The result is an approximation of the original scene, reconstructed from minimal measurements.

Besides the reconstruction method discussed above, it has been proven that the Differential Ghost Imaging (DGI) algorithm [35] is more robust against illumination fluctuation, thus generating better reconstructions. DGI introduces a corrected signal $D_{i}$,

(3) $$D_{i}=B_{i}-\frac{\langle B_{i}\rangle }{\langle R_{i}\rangle }R_{i},$$

where $R_{i}$ is the total intensity of the speckle pattern $I_{i}\left(x,y\right)$. This correction reduces the effect of global light fluctuations and background noise.

The DGI reconstruction is then expressed as

(4) $$G\left(x,y\right)=\langle D_{i}I_{i}\left(x,y\right)\rangle -\langle D_{i}\rangle \langle I_{i}\left(x,y\right)\rangle$$

This DGI-based reconstruction yields improved visual quality and structural consistency, which is particularly beneficial for downstream classification tasks based on SPI data. In addition to the basic concepts of SPI, we will further discuss two specific aspects of SPI in atmospheric turbulence.

*(A)*  *Learnable Measurement Matrix*

This paper introduces a learnable sampling matrix, $\Phi_{R}$, integrated into neural network training with end-to-end optimization.

In our framework, $\Phi_{R}$ is initialized using a Xavier normal distribution to ensure proper gradient flow and faster convergence. The initialization process is follows:

(5) $$\begin{array}{c} \Phi_{R}∼N\left(0,\frac{2}{n_{\mathrm{in}}+n_{\mathrm{out}}}\right) \end{array}$$

Here, $n_{\mathrm{in}}$ and $n_{\mathrm{out}}$ represent the input and output dimensions. This helps to prevent vanishing or exploding gradients during training.

After initialization, the matrix $\Phi_{R}$ is applied to the input image via convolution, allowing the network to learn optimal low-dimensional encoding while preserving important features. Throughout training, $\Phi_{R}$ is optimized along with the network parameters.

Because $\Phi_{R}$ is optimized, it can selectively capture critical image information, in contrast to static, random matrices used in traditional compressed sensing.

The dynamic adaptability of $\Phi_{R}$ improves classification accuracy and system robustness to noise, making it more efficient than pre-defined sampling matrices in challenging imaging environments.

To quantitatively describe the compression introduced by $\Phi_{R}$, we define the sampling rate sr as the ratio of the number of measurements *M* to the number of pixels *N* in the original two-dimensional scene:

(6) $$sr=\frac{M}{N},\;N=H\times W$$

where *H* and *W* are the height and width of the scene, respectively. For example, for a 128×128 image (N=16,384), a sampling rate of 3% corresponds to only M=492 learned projections.

Unlike fixed, randomly-generated sampling matrices used in traditional compressed sensing, the learnable matrix $\Phi_{R}$ dynamically adapts during training, selectively capturing the most informative features from the input. This adaptability leads to improved classification accuracy and robustness against noise, making the system significantly more effective in real-world imaging scenarios.

*(B)*  *Atmospheric turbulence*

Atmospheric turbulence significantly affects long-distance optical signal transmission, especially in remote sensing. It arises from fluctuations in the air’s refractive index caused by variations in temperature, pressure, and other environmental factors. These fluctuations induce random phase distortions in the propagating optical wavefront, leading to image blurring and degradation of spatial resolution.

To accurately model atmospheric turbulence, we employ a phase screen simulation based on the power spectrum inversion method [36]. This approach generates a phase distortion map representing the turbulence effect on the optical wavefront.

The power spectral density (PSD) of the atmospheric turbulence phase fluctuations is given by

(7) $$PSD_{\phi }\left(\kappa \right)=0.023r_{0}^{-5/3}\frac{\exp\left(-{\left(\frac{\kappa }{\kappa_{m}}\right)}^{2}\right)}{{\left(\kappa^{2}+\kappa_{0}^{2}\right)}^{11/6}}$$

where

- κ denotes the spatial frequency (rad/m), representing the inverse of spatial scale of turbulence eddies;
- $r_{0}$ is Fried’s parameter (m), describing the effective coherent length of the turbulence (a smaller $r_{0}$ means stronger turbulence);
- $\kappa_{m}=\frac{5.92}{l_{0}2\pi }$ is the inner scale cutoff frequency related to the smallest eddy size $l_{0}$;
- $\kappa_{0}=\frac{1}{L_{0}}$ is the outer scale cutoff frequency corresponding to the largest turbulence eddy size $L_{0}$.

The atmospheric turbulence phase screen AT is computed by

(8) $$AT=F^{-1}\left\{H\cdot \sqrt{PSD_{\phi }\left(\kappa \right)}\cdot \Delta f\right\}$$

where

- *H* is a complex Gaussian random matrix with zero mean and unit variance, representing random turbulence phase fluctuations;
- $\sqrt{PSD_{\phi }\left(\kappa \right)}$ shapes the spatial frequency content according to the turbulence model;
- Δf is the frequency sampling interval;
- $F^{-1}$ denotes the inverse Fourier transform, which converts the frequency-domain representation into a spatial-domain phase screen.

This phase screen modulates the phase of the incident optical wavefront to simulate the distortions caused by atmospheric turbulence. The resulting complex field captures both amplitude and phase variations induced by the turbulent atmosphere.

**Figure 3:**
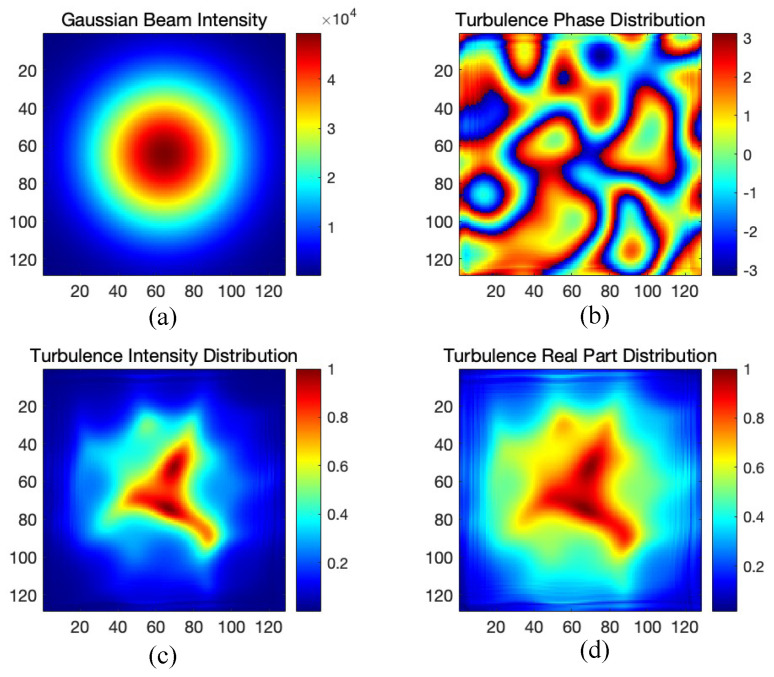
The simulation results of atmospheric turbulence: (**a**) Gaussian beam intensity before turbulent atmosphere, (**b**) turbulence-distorted field intensity, (**c**) turbulence distribution intensity, and (**d**) turbulence distribution in the real domain.

Figure 3 illustrates the simulation results: (a) shows the Gaussian beam intensity before turbulence, (b) depicts the phase distribution of the turbulence-distorted field, (c) presents the intensity distribution after turbulence, and (d) shows the real part of the turbulence-distorted field, providing a comprehensive visualization of turbulence effects.

### 3.2. Classification Network Hybrid-CTNet

In this section, we design a hybrid classification network Hybrid-CTNet tailored for sparse, turbulence-distorted single-pixel measurements. To balance local detail extraction and global semantic modeling, we propose a stage-wise (N+1)×L hybrid strategy, integrating convolutional blocks and Transformer modules.

*(A)*  *Hybrid Strategy*

As discussed in the Introduction section, recent efforts to combine Convolutional Neural Networks (CNNs) and Transformers use convolutional blocks in the early stages to extract local features and stack Transformer blocks in the final one or two stages to capture long-range dependencies and the global context. This approach has performance difficulties when it comes to complex downstream tasks like segmentation and detection. This is because such tasks require detailed features from all layers of the network, not just the final stage. The limited ability of traditional hybrid models, where Transformer blocks are only added in the last few layers, results in sub-optimal performance.

In remote sensing applications, this issue becomes even more pronounced. Remote sensing tasks often involve interpreting large, high-dimensional images, such as satellite or aerial imagery, where both fine-grained local features and global spatial context are crucial for accurate object detection and segmentation. Traditional CNN-based approaches may excel at local feature extraction, but they struggle to capture global relationships in large, complex scenes, which are often affected by environmental factors, scale variations, and clutter. Consequently, relying solely on convolutional layers may lead to incomplete feature representations, hindering the system’s ability to understand large-scale patterns within the scene.

**Figure 4:**
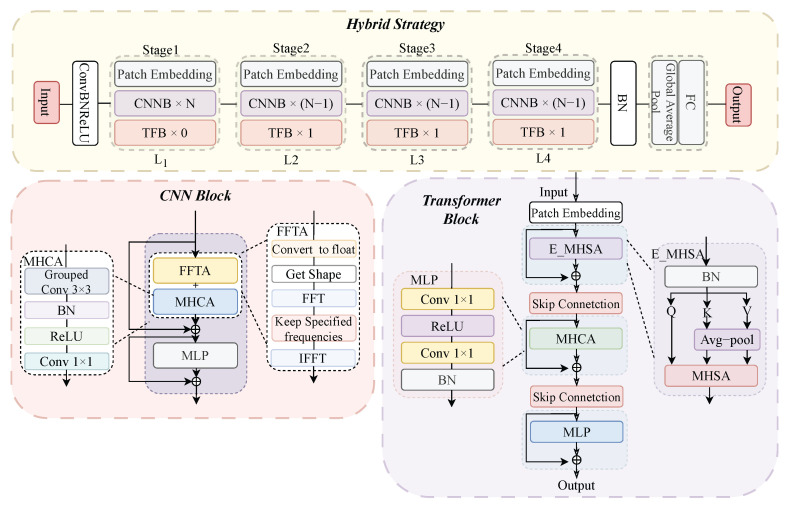
Hybrid strategy for object classification network.

To address this challenge, our study proposes a novel hybrid strategy that creatively integrates convolutional blocks and Transformer blocks using a (N+1)×L paradigm. This innovative approach enables the model to dynamically adjust the proportion of Transformer blocks at each stage of the network, effectively enhancing the model’s performance in downstream tasks like segmentation and detection. Specifically, we stack *N* convolutional blocks followed by a Transformer block in each stage, as shown in Figure 4. The key advantage of this design is that the Transformer block is placed at the end of each stage, which allows the model to capture global representations early on, even within the shallow layers of the network.

*(B)*  *The Convolutional block*

  The Convolution Block is an advanced neural network module combining Patch Embedding, Frequency Feature Transformation Attention (FFTA), and Multi-Head Channel Attention (MHCA). First, Patch Embedding downsamples and convolves the input to prepare feature maps. The feature maps then enter the FFTA module, which transforms spatial features into the frequency domain, retains key frequency components, and reconstructs refined features via inverse FFT. These frequency-enhanced features pass through MHCA, applying Multi-Head Channel Attention to emphasize important channels. Finally, the output is further transformed by a Multi-Layer Perceptron (MLP).

Note that FFTA operates in the latent feature space, not on reconstructed images, learning to highlight structurally discriminative components without direct spatial access.

This combination effectively integrates spatial and frequency information, enhancing feature expressiveness and model performance on complex tasks.

*(B-1)*  *Frequency Attention*

Frequency Attention improves the module’s focus on relevant frequency components. The feature map is transformed from the spatial to the frequency domain using Real FFT (RFFT), decomposing the input into frequency components. It then retains only a selected frequency range defined by freqdim, filtering out irrelevant or noisy frequencies. This helps the network capture important frequency-domain features, boosting representation quality and downstream task performance.

*(B-2)*  *Multi-Head Convolutional Attention (MHCA)*

MHCA enhances local feature extraction through grouped convolutions, BatchNorm, and ReLU, capturing spatial details across scales and contexts. The multi-head design enables simultaneous attention to different feature subspaces for effective local representation learning. MHCA’s efficient use of grouped and point-wise convolutions maintains inference speed.

Together with Frequency Attention, which filters key frequency components, MHCA enriches both spatial and frequency representations, improving model accuracy in complex tasks.

*(C)*  *The Transformer Block*

Transformer blocks excel at capturing low-frequency signals for global information (such as shape and structure), but can degrade high-frequency details such as local textures. As the human visual system requires a balance of different frequency bands, Transformer blocks must mix multi-frequency signals to enhance overall feature extraction.

The Transformer Block integrates attention mechanisms with an MLP and includes downsampling, normalization, and linear transformations. It enhances feature extraction by combining global and local attention, improving stability and efficiency through modular design. The Multi-Head Convolutional Attention (MHCA) and Efficient Multi-Head Self Attention (E_MHSA) modules work together to mix high- and low-frequency signals, with the MLP further refining features. Moreover, the block uses BatchNorm and ReLU layers, which, compared to traditional Transformer components, capture multi-frequency information efficiently and improve performance.

Among the several modules in the Transformer Block, we want to further discuss E_MHSA. Efficient Multi-Head Self Attention (E_MHSA) enhances computational efficiency by optimizing the multi-head self-attention mechanism. It consists of linear layers to generate queries (q), keys (k), and values (v) and an attention mechanism that computes dot products and applies a softmax function to generate attention weights. Reduction pooling is applied when the sr_ratio is greater than 1 to lower feature map resolution and reduce computational load, while batch normalization and projection layers ensure numerical stability. The advantage of E_MHSA lies in its ability to increase the efficiency of handling high-resolution inputs by reducing computational and memory overheads while retaining the global information extraction capabilities of the multi-head self-attention mechanism.

*(D)*  *Loss Function*

To accommodate soft-label supervision during training (e.g., due to Mixup augmentation), we adopt SoftTargetCrossEntropy loss, which generalizes the standard cross-entropy to handle probabilistic labels. For a training sample *i*, the loss is computed as

(9) $$L_{i}^{\mathrm{train}}=-\sum_{c=1}^{C}y_{ic}\log\left(\frac{e^{s_{ic}}}{\sum_{j=1}^{C}e^{s_{ij}}}\right)$$

where $s_{i}\in R^{C}$ denotes the model logits and $y_{i}\in {\left[0,1\right]}^{C}$ is the soft target label.

During validation and testing, where labels are one-hot encoded, we use the standard cross-entropy loss,

(10) $$L_{i}^{\mathrm{val}}=-\log\left(\frac{e^{s_{iy_{i}}}}{\sum_{j=1}^{C}e^{s_{ij}}}\right),$$

where $y_{i}\in \left\{1,\ldots ,C\right\}$ is the true class index.

The loss is averaged over all samples in the batch and backpropagated through both the classification network and the learnable sampling matrix Φ, which is treated as a trainable parameter. This end-to-end optimization allows the sampling pattern to evolve into a task-adaptive measurement operator that maximizes classification performance under turbulent conditions.

We further investigate the filtering characteristics of the learned sampling matrix in the frequency domain (see Section 4.3 (D)), revealing its ability to suppress turbulence-sensitive components and retain task-relevant features.

## 4. Simulated Experiments and Discussion

In this section, we conduct comprehensive simulation experiments to evaluate the effectiveness of our image-free classification framework under various sampling rates and imaging conditions. We first assess classification performance on standard datasets (e.g., MNIST, Fashion-MNIST), focusing on performance under different sampling rates. Then, we evaluate our model in a simulated remote sensing scenario with varying turbulence levels and scene complexities.

To further validate the advantages of our method, we compare it against both image-free and reconstruction-based classification pipelines under identical measurement conditions. In addition, frequency-domain and ablation analyses are conducted to reveal the internal mechanism and contribution of each component in our framework.

The end-to-end coding and decoding learning framework implements illumination optimization through the corresponding network, thus providing high acquisition and classification efficiency. When training the network, we use the Adamw solver for gradient optimization with weights decaying to 1 × 10^−4^.

### 4.1. The Datasets

*(A)*  *MNIST and Fashion-MNIST without turbulence*

The MNIST dataset contains 60,000 training and 10,000 test grayscale images of handwritten digits (28 × 28 pixels). It serves as a basic benchmark for classification. Fashion-MNIST has the same size but includes 10 fashion categories with more complex patterns and greater intraclass variability.

**Figure 5:**
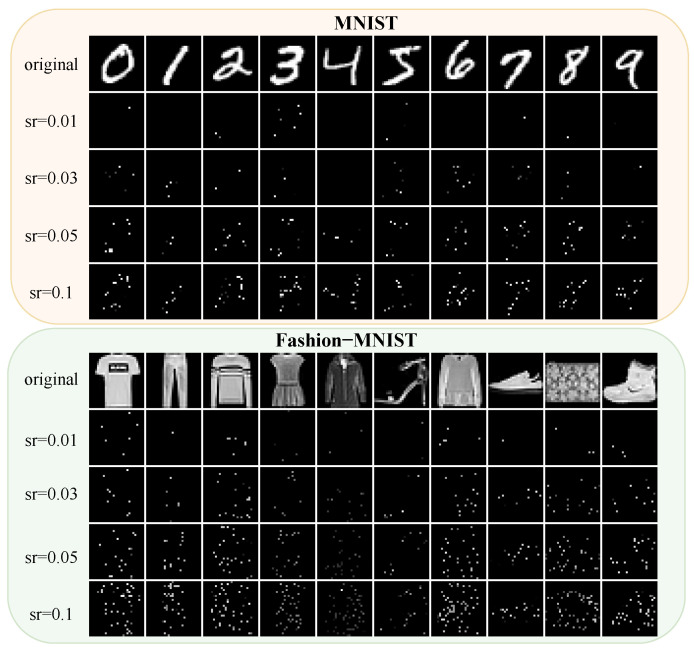
The reconstruction results of the MINIST and Fashion-MINIST datasets at sampling rates of 0.01, 0.03, 0.05, and 0.1, respectively.

These datasets allow for thorough benchmarking and demonstrate our method’s robustness, especially under low sampling rates common in applications like remote sensing and surveillance. As shown in Figure 5, at low sampling rates (sr), reconstructions barely reveal the objects. However, we can still classify objects with high accuracy using our network.

*(B)*  *UCM-SPI, MWPU-SPI and DOTA-SPI with turbulence*

To evaluate the performance of our proposed network, we conducted a comprehensive assessment using both remote sensing and traditional datasets. The NWPU-SPI VHR-10 dataset contains 10 classes of remote sensing scene images (700 images per class, 128 × 128 pixels), including objects such as airplanes, ships, oil tanks, baseball fields, tennis courts, basketball courts, athletic fields, ports, bridges, and vehicles. The UCM-SPI dataset consists of 21 ground target classes (100 images per class, 128 × 128 pixels), including agricultural areas, airplanes, baseball fields, beaches, urban building complexes, jungles, dense residential areas, forests, highways, golf courses, ports, intersections, central residential areas, mobile home parks, overpasses, parking lots, rivers, runways, sparse residential areas, oil storage tanks, and tennis courts. Both datasets feature multi-scale objects and background interference, providing a robust platform for evaluating the model’s generalization ability in remote sensing.

To assess our system’s robustness under atmospheric turbulence, we incorporated turbulence simulations with varying intensities (10 and 100 layers) into our dataset, reflecting real-world weather scenarios. This allowed us to evaluate the system’s performance under different levels of environmental distortion, mimicking the challenges faced in practical remote sensing tasks like environmental monitoring, weather prediction, and surveillance.

**Figure 6:**
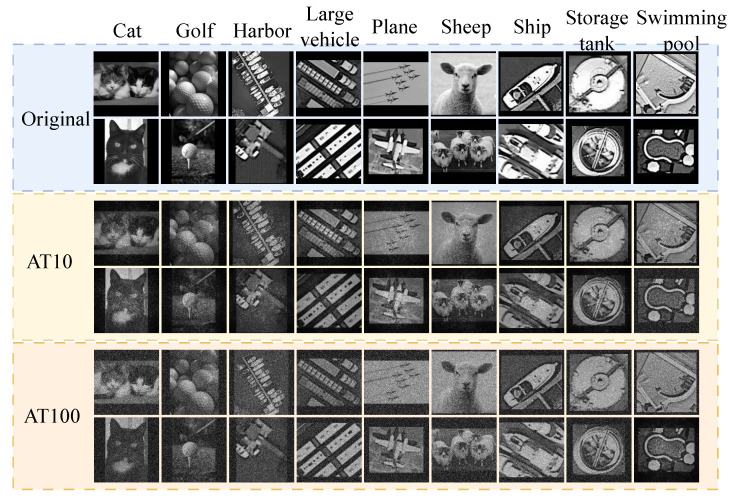
Our datasets.

We also used the DOTA-SPI (Dataset for Object Classification in Aerial Images) dataset, which is widely used for remote sensing tasks. We cropped 128 × 128 pixel-sized image patches from the DOTA-SPI dataset to create a custom dataset with nine distinct object categories across 20,480 images, combining aerial scenes from DOTA-SPI and various scene types from PASCAL VOC. To better evaluate the performance of the network modules, we mixed these two datasets, as shown in Figure 6. This diverse dataset allows us to assess the performance of the network in different domains, from remote sensing to traditional object classification tasks.

### 4.2. Image-Free Classification Comparison Using MNIST and Fashion-MNIST Without Turbulence

To ensure fair comparisons with existing methods and to demonstrate the practical applicability of our network, we validated our approach on two widely used public datasets—MNIST and Fashion-MNIST—which serve as standard benchmarks for image classification tasks. All compared methods follow the reconstruction-free (image-free) paradigm, directly mapping compressed measurements to classification outputs without intermediate image recovery.

**Figure 7:**
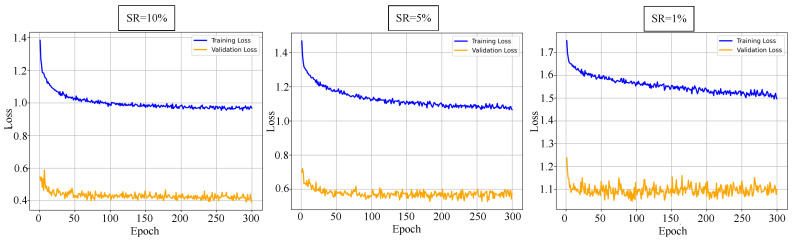
Training and validation loss curves under varying sampling rates and turbulence conditions.

Before going further with the comparison of different methods, we first evaluate the convergence behavior of our image-free classification model under different compression levels. We plot the training loss curves on the Fashion-MNIST dataset at sampling rates of 1%, 5%, and 10%. As shown in Figure 7, the network remains trainable even under extremely low sampling rates (1%), with stable convergence across all settings. Higher sampling rates lead to faster convergence and lower final loss values, indicating that increased measurement redundancy improves optimization efficiency and learning capacity.

Table 1 shows the classification accuracy (%) of our method versus the reference method under different sampling rates for both datasets. No noise is added in the measurements. For the MNIST dataset, our method consistently outperforms the reference method. For example, at a sampling rate of 0.01, our method achieves 98.7% accuracy compared to 90.4% for the reference, while at a sampling rate of 0.10, the accuracies are 99.4% and 98.5%, respectively. Similarly, for the Fashion-MNIST dataset, our approach achieves 89.8% accuracy at a sampling rate of 0.01 versus 81.8% for the reference, and 91.3% versus 88.1% at a sampling rate of 0.10. These results confirm the effectiveness of our approach, particularly under conditions of limited data availability.

In the single-pixel imaging-free target classification task, the system is prone to environmental interference, which can lead to measurement inaccuracies. To test the robustness of our proposed method, we introduced salt-and-pepper noise at 10.0%, 13.0%, and 15.0% levels into the MNIST and Fashion-MNIST test datasets. Salt-and-pepper noise randomly zeroes or saturates pixels, simulating abrupt electromagnetic interference or sensor failures [33]. Unlike Gaussian noise, which causes a mild global disturbance, salt-and-pepper noise disrupts local features more severely. In our implementation, salt-and-pepper noise was directly added to the measurement values. This simulates interference occurring during the sensing process, which is more consistent with real-world acquisition scenarios.

At a sampling rate of 2.9%, the performance of the proposed method was compared with three alternative methods—NHDM (Naturally ordered Hadamard), WSHDM (Walsh Hadamard), and CCHDM (Cake-cutting Hadamard)—using an SVM (Support Vector Machine) classifier.The experimental results indicate in Table 2, as the salt-and-pepper noise ratio increases, the acquired data from all methods contain more interference, leading to a decline in recognition accuracy. However, the reduction in accuracy for the proposed method is notably less severe. For instance, at a 13% noise level, the proposed method maintains a relatively high recognition accuracy on both the MNIST and Fashion MNIST datasets, while the alternative methods exhibit a more pronounced degradation in performance. Based on these observations, we preliminarily conclude that the proposed single-pixel imaging-free target classification method possesses robust anti-interference capabilities.

### 4.3. Image-Free Classification Comparison Using UCM-SPI, MWPU-SPI, and DOTA-SPI with Turbulence

*(A)*  *Training Dynamics and Convergence Analysis.*

**Figure 8:**
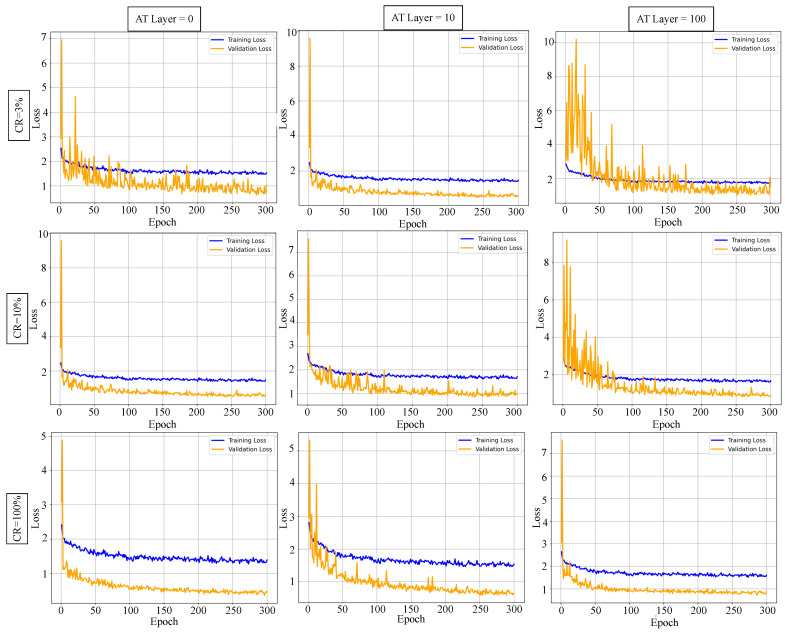
Training and validation loss curves under varying sampling rates and turbulence conditions.

To enhance transparency, we analyze training dynamics by visualizing the training and validation loss curves under different turbulence levels (0-layer, 10-layer, and 100-layer) and sampling rates (3%, 10%, and 100%) on the NWPU-SPI dataset, as shown in Figure 8. The loss steadily decreases throughout 300 epochs, reflecting the stability and convergence of the Hybrid-CTNet. Even under severe turbulence and sparse sampling (e.g., 3%, 100 layers), the loss exhibits a smooth declining trend, demonstrating the robustness of our framework.

Further analysis reveals that both the atmospheric turbulence (AT) layer number and sampling rate (sr) significantly affect the final convergence loss. Specifically, the loss increases consistently as the AT layer count rises from 0 to 100, highlighting the increasing difficulty of accurate reconstruction under complex distortion. Meanwhile, higher sr values (e.g., from 3% to 100%) substantially reduce the loss, confirming that more measurements improve signal recovery performance. Notably, under low-sr conditions, the influence of turbulence becomes more pronounced, with steeper loss curves and a higher final error, whereas high-sr settings mitigate turbulence-induced degradation. These results underscore the dual role of sampling adequacy and turbulence modeling in achieving reliable convergence in turbulent compressive sensing.

*(B)*  *Image-Free Classification with ViT.*

To further validate the effectiveness of our design under the image-free paradigm, we introduce a ViT-based reconstruction-free baseline for direct comparison. In this setting, the same learned sampling matrix is used to generate compressed measurements, which are then fed directly into a lightweight Vision Transformer without any image reconstruction. As shown in Table 3, our model significantly outperforms the image-free ViT baseline across multiple datasets, sampling rates, and turbulence levels.

Under challenging conditions such as low sampling rates and severe atmospheric turbulence, the performance of the ViT baseline drops sharply due to its limited ability to extract robust representations from distorted and sparse measurements. In contrast, our Hybrid-CTNet architecture remains highly effective, benefiting from spatially aware convolutional priors and Transformer-based global modeling. This performance gap highlights the superiority of our design in turbulent, low-information environments. Moreover, our model achieves these results with substantially fewer parameters and lower computational cost, making it particularly suitable for resource-limited deployment scenarios such as satellite-based or airborne sensing platforms.

*(C)*  *Ablation Study of Modules.*

The ablation study, as shown in Table 4, evaluates the performance of different module combinations on three datasets, NWPU-SPI, UCM-SPI, and DOTA-SPI, with varying sampling rates (0.5, 0.1, 0.03) and turbulence layer levels (0, 10, 100). The results highlight the contribution of each component and demonstrate the change in the model’s performance under different experimental conditions.

1. Learnable Sampling Matrix + CNNB (CNN Block only): The combination of the learnable sampling matrix and CNNB shows varying performance across the three datasets. For the NWPU-SPI dataset, the accuracy is 75.7% at a sampling rate of 0.03 and 100 turbulence layers. For the UCM-SPI dataset, performance is slightly higher, but still limited, while for the DOTA-SPI dataset, the accuracy improves to a higher level at a sampling rate of 0.5 but still suffers under lower sampling rates and higher turbulence. The learnable sampling matrix helps to compress the data, but CNNB alone lacks the ability to effectively capture global context, especially under challenging conditions with sparse data and strong turbulence. As the sampling rate and turbulence level increase, the performance becomes increasingly suboptimal due to the model’s reliance on local features only.

2. Learnable Sampling Matrix + TFB (Transformer Block only): Combining the learnable sampling matrix with the Transformer-based model (TFB) results in a significant drop in performance, especially under low sampling rates and high turbulence. For example, based on the NWPU-SPI dataset with a sampling rate of 0.03 and 100 turbulence layers, the accuracy is only 55.6%. Based on the UCM-SPI dataset, the accuracy also drops significantly under similar conditions, and based on the DOTA-SPI dataset, the model fails to achieve high performance under lower sampling rates and turbulence layers. TFB excels in capturing long-range dependencies but lacks the ability to effectively handle local feature extraction, which is critical for these scenarios. The model struggles with sparse data and distorted measurements, leading to its poor performance.

3. CNNB + TFB: The hybrid combination of CNNB and TFB shows improvement across all datasets compared to the previous two configurations. Based on the NWPU-SPI dataset, the accuracy reaches 63.4% at a sampling rate of 0.03 and 100 turbulence layers. Based on UCM-SPI, accuracy is also higher compared to the previous models but still does not reach optimal performance. Based on the DOTA-SPI dataset, the model performs relatively better at the higher sampling rate (0.5) and lower turbulence levels. This hybrid approach allows CNNB to handle local feature extraction while TFB captures the global context, but without the learnable sampling matrix, the model still suffers from data redundancy, limiting its performance under more challenging conditions.

We also evaluated the impact of different optimizers. We compared Stochastic Gradient Descent (SGD) and AdamW within our proposed classification network. As shows in Table 5, AdamW outperformed SGD in terms of convergence speed, model accuracy, and training efficiency. Its adaptive learning rate adjustment and weight decay integration improved parameter optimization and reduced overfitting, resulting in faster convergence and better generalization. In contrast, while SGD was simple and easy to implement, it suffered from slower convergence and required manual tuning of learning rates and momentum, which was time-consuming and suboptimal. Our results consistently showed that AdamW provided more efficient and effective training, making it the preferred optimizer for our task.

*(D)*  *Frequency-Domain Analysis of the Learned Sampling Matrix.*

To understand the mechanism that enables our learned sampling matrix $\Phi_{R}$ to maintain robustness under strong turbulence, we performed a frequency-domain statistical analysis under the extreme case of 100-layer turbulence and compared it with a randomly generated binary matrix under an identical sampling rate (sr=3%).

This analysis is inspired by the convolution theorem, which states that convolution in the spatial domain corresponds to multiplication in the frequency domain. In single-pixel imaging, the measurement process can be viewed as an inner-product operation between the scene and sampling patterns, effectively acting as a frequency filter. Therefore, examining $\Phi_{R}$ in the frequency domain reveals how it selects and emphasizes specific spectral components.

**Figure 9:**
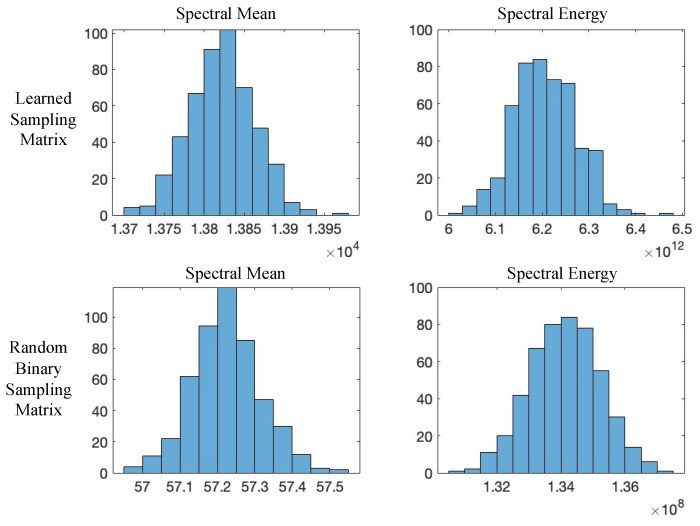
Frequency-domain comparison of learned (top) vs. random binary (bottom) sampling matrices under 100-layer turbulence.

Each histogram in Figure 9 shows the statistical distribution of frequency magnitudes (horizontal axis) across all sampling patterns, with the vertical axis indicating the number of patterns falling into each frequency–magnitude bin. This visualization enables a direct comparison of frequency-domain characteristics between the learned and random sampling strategies.

**(1) Spectral Mean:** The learned sampling matrix exhibits a significantly higher average spectral magnitude (13,822) compared to the random baseline (57.22), indicating that it concentrates more energy in informative spectral regions and yields more stable measurements.

**(2) Spectral Energy:** The total spectral energy of the learned matrix is $6.2\times 10^{12}$, substantially greater than $1.3\times 10^{8}$ for the random matrix. This suggests better energy preservation across frequencies, which is critical for maintaining signal integrity under turbulence.

**(3) Spectral Coverage:** The learned matrix exhibits broader frequency coverage, spanning both low and high frequencies. This wide spectral span enables it to capture richer scene structures while suppressing low-frequency turbulence artifacts.

**(4) High-Frequency Compactness:** We further computed high-frequency compactness, defined as the proportion of spectral energy lying outside a centered 32×32 low-frequency window. The learned matrix achieves a compactness of 0.59, versus 0.47 for the random matrix, confirming its ability to retain high-frequency structural information important for turbulence-resilient recognition.

Together, these findings demonstrate that the learned sampling matrix trained under 100-layer turbulence acts as a turbulence-adaptive frequency selector. By encoding a broader and more informative frequency spectrum, it enhances both measurement quality and downstream classification performance in severely degraded imaging scenarios.

### 4.4. Comparisons with Reconstruction-Dependent Methods

*(A)*  *Comparisons with YOLOv11, MobileNetV3, and ViT.*

**Figure 10:**
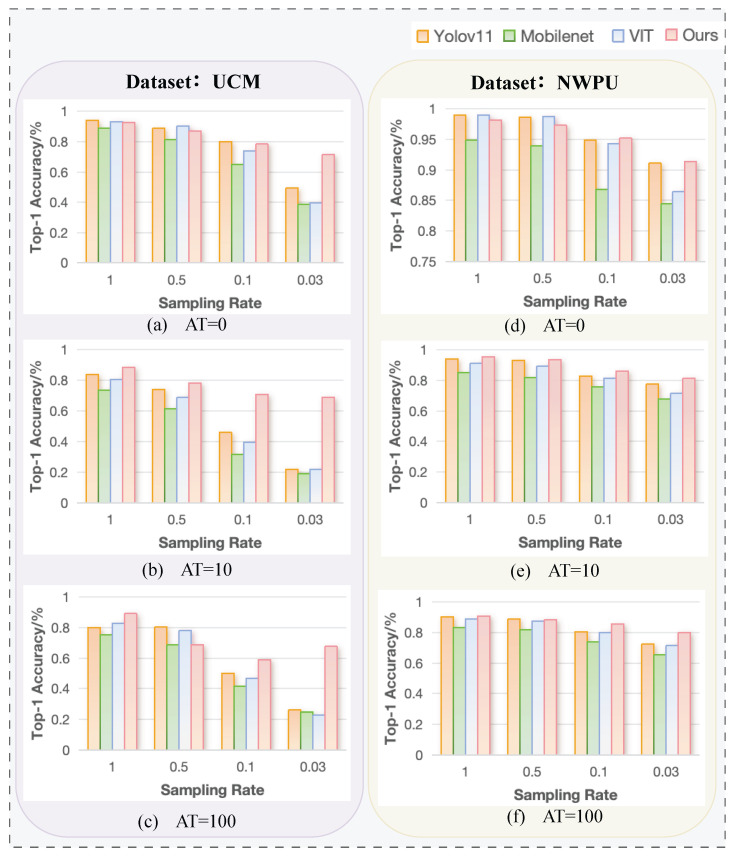
Top 1 (%) accuracy of different methods on the UCM-SPI-SPi and NWPU-SPI-SPU datasets for object classification.

As shown in Figure 10, the proposed method (Hybrid-CTNet) significantly outperforms several state-of-the-art models based on both the UCM-SPI and NWPU-SPI remote sensing datasets under varying turbulence conditions. The models compared include YOLOv11, MobileNetV3 [38], and ViT [39]. Among these three networks, YOLOv11 is a well-known real-time object detection model that prioritizes speed and efficiency. However, its performance degrades significantly under high turbulence and low sampling rates. MobileNetV3 is designed for lightweight applications and performs well in standard settings. However, it struggles in remote sensing tasks, especially when faced with sparse data and turbulence. ViT (Vision Transformer) excels in traditional image classification tasks but its performance drops when the sampling rate is low or turbulence is high due to its reliance on pixel-level feature extraction.

Note that YOLOv11, MobileNetV3, and ViT are networks based on 2D images. Thus, before applying these models, the input images are first reconstructed from single-pixel measurements using the differential ghost imaging (DGI) algorithm. The resulting reconstructions from Equation (4) are then fed into YOLOv11, MobileNetV3, and ViT for performance evaluation under turbulent conditions.

In our experiments, we allocated 90% of the images for training and the remaining 10% for testing to ensure robust evaluation. As shown in Figure 10, our method consistently outperforms YOLOv11, MobileNetV3, and ViT on both the UCM-SPI and NWPU-SPI datasets. For example, on the NWPU-SPI dataset with AT = 100, our method achieves 84.1% accuracy. The key advantage of our approach becomes more apparent as turbulence intensity increases and sampling rates decrease. Particularly in the context of non-image object detection with a sampling rate as low as 3%, our method achieves 91.69% accuracy, demonstrating its effectiveness even with minimal data. At higher sampling rates, the system exhibits even better robustness—accuracy improves across all turbulence levels, reaching 96.68% in the 0-layer case and remaining around 95% for both the 10-layer and 100-layer turbulence conditions. These values can be clearly observed in Figure 10, which summarizes the accuracy results under different conditions. The results confirm that our hybrid convolutional-Transformer network maintains high accuracy even in challenging conditions and is less sensitive to turbulence intensity at higher sampling rates, making it well-suited for real-world remote sensing applications.

Table 6 compares the model sizes and computational costs of different classification networks. Our model has the fewest parameters (1.8 million), which is much smaller than ViT (85 million), YOLOv11n (9.4 million), and MobileNetV3 (4.2 million). Although our FLOPs ($7.6\times 10^{7}$) are slightly higher than MobileNetV3’s ($7.2\times 10^{6}$), they are still far lower than those of ViT ($5.6\times 10^{9}$) and YOLOv11n ($2.2\times 10^{10}$). Notably, our model does not require an image restoration module, unlike the others. This makes our approach more efficient and suitable for resource-limited scenarios like satellite-edge computing.

In general, the accuracy of YOLOv11, MobileNetV3, and ViT is highly dependent on the quality of image reconstruction, which can be severely affected by factors such as turbulence and weather conditions. As a result, their performance degrades under low sampling rates and high turbulence, whereas our approach remains robust and effective, as it does not rely on image reconstruction but directly classifies sparse measurements. In addition, YOLOv11, MobileNetV3, and ViT rely on random binary pattern sampling followed by image reconstruction for classification, while our method employs a learnable sampling matrix for direct image-free classification.

*(B)*  *Comparisons with Ref. [34].*

Table 7 presents a quantitative comparison between our method and Ref. [34] under varying sampling rates (50%, 10%, and 3%) and turbulence layer counts (0, 10, and 100). Although Ref. [34] also considers the effect of turbulence and addresses a classification task, it adopts a two-stage pipeline, first reconstructing the image from Hadamard-coded measurements, followed by classification based on the reconstructed image. In contrast, our method performs direct classification from compressed measurements, bypassing image reconstruction entirely. In addition, the sampling matrix in Ref. [34] is a Hadamard matrix, which is not designed for classification using SPI, while our method jointly designed the sensing matrix for classification.

Despite these methodological differences, we report the results from Ref. [34] for reference. As shown, our method consistently outperforms Ref. [34] across all conditions. For instance, at a low sampling rate of 3% with 100 turbulence layers, our method achieves 0.801 classification accuracy, significantly higher than the result using the method in Ref. [34]. This performance gap is especially evident under severe turbulence and low sampling rates, demonstrating the robustness of our model in extremely degraded conditions.

These results validate that our end-to-end classification framework—featuring jointly optimized sensing and inference—is not only more efficient (no reconstruction required) but also more resilient to turbulence, supporting its potential for real-time, resource-constrained remote sensing applications.

## 5. Optical Experiments

**Figure 11:**
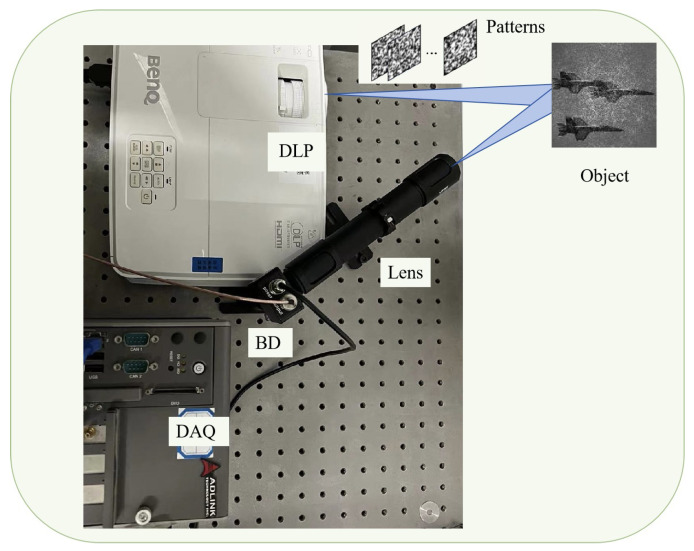
The optical experimental system for single pixel imaging.

After validating the feasibility and effectiveness of the designed system, we proceeded with data collection based on the experimental setup depicted in Figure 11. Our optical experiment platform for single-pixel imaging (SPI) was specifically designed to demonstrate the practical implementation and performance of the proposed system in real-world conditions [40]. The SPI apparatus consisted of several key components: a Digital Light Projector (DLP), a condenser lens, a barrel-shaped detector (BD), a personal computer (PC), and a data acquisition system (DAQ).

### 5.1. The Optical Experimental System

The DLP (BENQ-ED935) was used to project binary templates onto the target objects with a WXGA resolution of 1280 × 800 pixels and a brightness of 1815 lumens, ensuring clear and high-quality images under varying environmental conditions. Its 60 Hz refresh rate ensured stability in the projected images during rapid sampling, which is essential for accurate measurements. A condenser lens system, with two lenses of focal length 26.5 mm, focused the reflected light onto the photodetector (DH-GDT-D020V), which has a broad spectral range of 400–1100 nm, ensuring efficient signal capture in different lighting conditions. The DAQ (ADLink-PCIe9814) sampled at 100 kSa/s, providing high-quality data acquisition. The processed signals were then transmitted to the PC for further analysis, where classification algorithms were applied to the data.

For the experimental setup, a learned sampling matrix was used. At a sampling rate of 0.03, only 492 samples are needed for a 128 × 128 object, greatly reducing data collection. We prepared a dataset of 300 samples from three classes (golf balls, aircraft, and cats), including single and combined objects with various poses, occlusions, and interference to test robustness. The targets were emulated from the DOTA-SPI dataset, and turbulence-degraded images were generated at different propagation distances by applying the turbulence phase screens described earlier. This method allowed the controlled simulation of atmospheric turbulence effects on the targets.

### 5.2. The Optical Experimental Results

**Figure 12:**
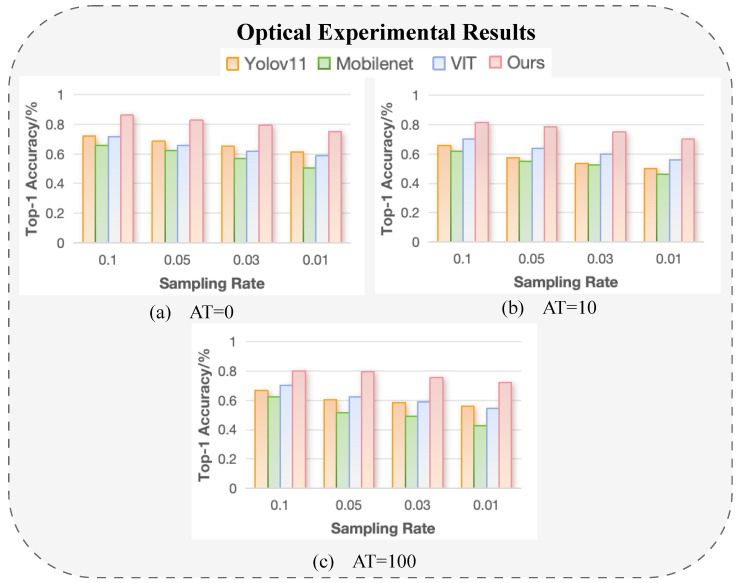
Top 1 (%) accuracy of different methods on the UCM-SPI and NWPU-SPI datasets for object classification.

To evaluate the classification performance under different sampling rates and turbulence levels, we conducted experiments at sampling rates of 10%, 5%, 3%, and 1% across three turbulence conditions (0-layer, 10-layer, and 100-layer). We compared MobileNetV3, ViT, YOLOv11, and our proposed method. Notably, the other three methods required image reconstruction using the differential ghost imaging (DGI) algorithm before classification and remained heavily dependent on the quality of the reconstructed images. Due to the inherent noise and system imperfections in the optical experimental setup, all methods showed a decline in accuracy as the sampling rate decreased. However, as shown in Figure 12, our method consistently outperformed the others across all sampling rates and turbulence levels, demonstrating superior robustness and reliability. Subfigures (a), (b), and (c) in Figure 12 correspond to turbulence levels of 0 layers, 10 layers, and 100 layers, respectively, with each summarizing the classification accuracy of the four methods under varying sampling rates.

While the classification results obtained from optical experiments are promising, they are lower than the simulation-based outcomes, particularly under extreme conditions such as strong turbulence or low sampling rates. This discrepancy primarily arises from real-world imperfections that are not present in simulation environments. In practice, the measurement quality is affected by several factors. First, the dynamic range and sensitivity of the photodetector and data acquisition system may limit the system’s ability to capture subtle intensity fluctuations, especially under weak modulation. Second, background ambient light introduces unwanted noise that superimposes on the structured illumination response, reducing the signal contrast received by the single-pixel detector. Third, fluctuations in the temperature of the light source or surrounding environment can cause temporal instability in the measurement signals. Lastly, insufficient brightness from the projector limits the modulation depth of the patterns projected onto the scene, further degrading the signal-to-noise ratio (SNR) of the captured 1D measurements.

Despite these real-world constraints, our proposed method demonstrates strong robustness and maintains a clear performance advantage over baseline methods, as evidenced in Figure 12. Even under significant turbulence and minimal sampling, the Hybrid-CTNet consistently outperforms reconstruction-based and non-learned approaches. This highlights the resilience of our end-to-end image-free classification pipeline to both algorithmic and hardware-level perturbations. The ability to sustain high accuracy across challenging conditions confirms the practical viability of our system for real-world single-pixel imaging tasks, particularly in scenarios with limited bandwidth, noisy measurements, or constrained computation.

## 6. Conclusions

In this paper, we proposed a turbulence-resilient, image-free object classification framework based on single-pixel imaging (SPI). By integrating a learnable sampling matrix with a hybrid CNN–Transformer backbone (Hybrid-CTNet), the system directly infers object categories from compressed 1D measurements, eliminating the need for image reconstruction. This design substantially reduces data volume and computation, enabling efficient perception in resource-limited remote sensing scenarios.

Extensive experiments demonstrate that our framework maintains high classification accuracy under extremely low sampling rates and strong optical turbulence, outperforming both reconstruction-based and other image-free methods. The system also exhibits good convergence behavior and strong generalization across different imaging conditions. Frequency-domain and ablation analyses further confirm the adaptability and effectiveness of each proposed module.

While promising, this method has limitations. The current evaluations are conducted under simulated or semi-controlled conditions, and its performance under fully dynamic real-world turbulence remains to be validated. The framework also assumes static scenes and stable illumination, which may restrict its application in rapidly changing environments. Additionally, although computationally lightweight, real-time deployment requires further progress in hardware acceleration and synchronization.

Future work will focus on the following: (i) incorporating dynamic turbulence models or real atmospheric measurements; (ii) extending the framework to handle scene motion and illumination changes; and (iii) developing end-to-end real-time implementations on embedded or photonic hardware for field deployment.

## Figures and Tables

**Table 1:** Classification accuracy (%) on the MNIST and Fashion-MNIST datasets.

Dataset	Method	0.01	0.03	0.05	0.10
MNIST	**Ref. [37]**	0.904	0.971	0.977	0.985
	**Ours**	0.987	0.989	0.991	0.994
Fashion-MNIST	**Ref. [37]**	0.818	0.862	0.876	0.881
	**Ours**	0.898	0.901	0.906	0.913

**Table 2:** Comparison of NHDM, WSHDM, CCHDM, and our model under sr = 2.9% when salt-and-pepper noise is added into measurements.

Dataset	Condition	NHDM	WSHDM	CCHDM	Our Model
MNIST	Noise-free	0.593	0.818	0.897	0.982
	Noise-10.0%	0.276	0.572	0.625	0.814
	Noise-13.0%	0.234	0.492	0.508	0.768
	Noise-15.0%	0.217	0.418	0.435	0.736
Fashion MNIST	Noise-free	0.760	0.714	0.808	0.901
	Noise-10.0%	0.659	0.640	0.754	0.763
	Noise-13.0%	0.609	0.602	0.710	0.722
	Noise-15.0%	0.582	0.574	0.682	0.676

**Table 3:** Comparison of reconstruction-free classification: our Hybrid-CTNet vs. the ViT baseline.

Sampling Rate (%)	50			10			3		
**Turbulence Layers**	0	10	100	0	10	100	0	10	100
**Dataset**	**NWPU-SPI**								
**Vit**	0.905	0.842	0.779	0.891	0.763	0.711	0.879	0.762	0.726
**Our Model**	0.973	0.938	0.883	0.952	0.862	0.857	0.914	0.815	0.801
**Dataset**	**UCM-SPI**								
**Vit**	0.562	0.445	0.348	0.446	0.376	0.25	0.413	0.357	0.227
**Our Model**	0.870	0.784	0.689	0.784	0.709	0.590	0.715	0.692	0.676
**Dataset**	**DOTA-SPI**								
**Vit**	0.909	0.864	0.837	0.871	0.826	0.778	0.863	0.822	0.768
**Our Model**	0.955	0.947	0.941	0.939	0.921	0.925	0.917	0.882	0.871

**Table 4:** Classification performance of different backbone configurations (CNNB, TFB, and CNNB + TFB) across datasets.

Sampling Rate (%)	50			10			3		
**Turbulence Layers**	0	10	100	0	10	100	0	10	100
**Dataset**	**NWPU-SPI**								
**CNNB**	0.957	0.916	0.841	0.934	0.844	0.801	0.891	0.775	0.757
**TFB**	0.828	0.712	0.689	0.788	0.670	0.647	0.770	0.578	0.556
**CNNB + TFB**	0.955	0.844	0.810	0.895	0.795	0.738	0.796	0.772	0.634
**Dataset**	**UCM-SPI**								
**CNNB**	0.857	0.763	0.682	0.787	0.633	0.546	0.680	0.461	0.422
**TFB**	0.401	0.319	0.294	0.364	0.288	0.263	0.302	0.253	0.227
**CNNB + TFB**	0.828	0.641	0.651	0.638	0.426	0.401	0.404	0.223	0.226
**Dataset**	**DOTA-SPI**								
**CNNB**	0.948	0.938	0.923	0.926	0.921	0.907	0.903	0.892	0.883
**TFB**	0.849	0.827	0.819	0.792	0.768	0.712	0.765	0.728	0.670
**CNNB + TFB**	0.968	0.945	0.928	0.959	0.941	0.907	0.932	0.887	0.401

**Table 5:** Classification accuracy (%) with different optimizers and conditions.

Optimizer Condition	NT, sr = 3%	NT, sr = 1	AT, sr = 3%	AT, sr = 1
**SGD**	0.909	0.962	0.878	0.947
**AdamW**	0.916	0.967	0.882	0.955

**Table 6:** Model metrics of classification network.

Network	Parameter Count	FLOPs	Restoration
**YOLOv11n**	$9.4\times 10^{6}$	$2.2\times 10^{10}$	✓
**ViT**	$8.5\times 10^{7}$	$5.6\times 10^{9}$	✓
**MobileNetV3**	$4.2\times 10^{6}$	$7.2\times 10^{6}$	✓
**Ours**	$1.8\times 10^{6}$	$7.6\times 10^{7}$	×

**Table 7:** Evaluating classification under turbulence: Hybrid-CTNet vs. Ref. [34].

Sampling Rate (%)	50			10			3		
**Turbulence Layers**	0	10	100	0	10	100	0	10	100
**Ref. [34]**	0.804	0.723	0.694	0.719	0.763	0.5	0.711	0.729	0.547
**Ours**	0.973	0.938	0.883	0.952	0.862	0.857	0.914	0.815	0.801

## Data Availability

The original contributions presented in the study are included in the article, further inquiries can be directed to the corresponding author.
